# The Thermo-Mechanical Response of GeTe under Compression

**DOI:** 10.3390/ma15175970

**Published:** 2022-08-29

**Authors:** Gilad Mordechai Guttmann, Shmuel Samuha, Reuven Gertner, Barak Ostraich, Shlomo Haroush, Yaniv Gelbstein

**Affiliations:** 1 Department of Materials Engineering, Ben-Gurion University of the Negev, P.O. Box 653, Beer-Sheva 8410501, Israel; 2 Nuclear Research Center Negev, P.O. Box 9001, Beer-Sheva 8419001, Israel; 3 Nuclear Engineering Department, University of California, 4153 Etcheverry Hall Berkeley, Berkeley, CA 94720, USA

**Keywords:** GeTe, compression, mechanical properties, crystallography, EBSD, geometrically necessary dislocations

## Abstract

Thermoelectric generators (TEGs) are devices capable of transforming heat energy into electricity and vice versa. Although TEGs are known and have been in use for around five decades, they are implemented in only a limited range of applications, mainly extraterrestrial applications. This is due to their low technical readiness level (TRL) for widespread use, which is only at levels of 3–5 approaching laboratory prototypes. One of the most setbacks in reaching higher TRL is the lack of understanding of the mechanical and thermo-mechanical properties of TE materials. Out of ~105,000 entries about TE materials only ~100 entries deal with mechanical properties, while only 3 deal with thermo-mechanical properties. GeTe-based alloys with varying other elements, forming efficient *p*-type thermoelectric materials in the 200 ÷ 500 °C temperature range, have been intensively researched since the 1960s and have been successfully applied in practical TEGs. Yet, their temperature-dependent mechanical properties were never reported, preventing the fulfillment of their potential in a wide variety of practical applications. The combined effects of temperature and mechanical compression of GeTe were explored in the current research by implementing novel quantitative crystallographic methods to statistically describe dislocation activity and modification of the micro-texture as inflecting by the testing conditions. It is suggested, through utilizing these methods, that the combined effect of compression and temperature leads to the dissolving of twin boundaries, which increases dislocation mobility and results in a brittle-to-ductile transition at ~0.45 of the homologous temperature.

## 1. Introduction

Thermoelectric generators (TEGs) are devices capable of transforming heat energy into electricity. As such, they have potential for use as green electrical energy sources in a wide range of applications where common producing methods are either unavailable or scarce—such as extraterrestrial vehicles. Furthermore, they can be attached to multiple applications for recovering waste residual heat and enhancing the overall efficiency of energy conversion systems. The TEGs consist of a large number of thermoelectric pairs constructed of sub-element with n-type and p-type thermo-elements, connected electronically to each other via conductor plates. The structural materials are semiconducting thermoelectric (TE) materials that are characterized by high Seebeck coefficients, low thermal conductivity, and high electrical conductivity. This set of physical properties is commonly expressed by a non-dimensional “quality factor” known as the figure of merit (ZT).

Developing a thermoelectric device with high efficiency and high operational reliability is complex due to the poor mechanical properties of known TE materials and low thermal expansion compatibility between TEG elements. TE materials may also exhibit thermal instability at a given service temperature range due to thermal-driven phase changes and thus will require either suitable isolating coatings, working in an inert environment at appropriate pressures, or adjusting the temperature range—thus lowering the overall efficiency. Additionally, soldering the thermoelectric material to the metal conductor plate serves as a point of failure mainly due to the degradation of the solder materials and poor compatibility between the solder and TE material. During service, mechanical stresses develop in the device as a result of static and dynamic mechanical forces that threaten the overall integrity of the construction materials [1,2,3].

In recent decades, many studies have been conducted to develop TE materials with as high a figure of merit as possible. At the same time, studies have been carried out, albeit significantly smaller in quantity, on the mechanical properties of TE materials [4,5]. Within this small group of research, the most published property (~92%) is hardness (by various techniques), with some of them pushing further and estimating the fracture toughness from hardness indents. The remaining research employed other mechanical testing techniques such as flexure and compression, while in only one article was tensile employed [6]. In the vast majority of studies, the properties of the materials were tested at room temperature rather than at the working temperature range at which they are expected to be used. Only four articles published mechanical data and measured properties at different temperatures than room temperature [6,7,8,9]. Therefore, measuring and understanding the mechanical properties of TE materials remains a gap of the research, while estimating and understanding their temperature dependence is a gap of knowledge.

One of the most researched and promising TE classes of materials used in TEGs are GeTe-based alloys with varying elements (which are part of the TAGS group), forming a series of efficient thermoelectric materials in the 200–500 °C temperature range. These materials have been intensively researched since the 1960s and have been successfully applied in several thermoelectric energy generators [10,11,12,13,14]. With increasing temperature, GeTe-based alloys are characterized by the phenomenon of phase transition from the rhombohedral to cubic structures.

The present study focused on the influence of temperature on the quasi-static mechanical behavior of the GeTe compound. A dedicated hot-compression apparatus was built, enabling measurements of the mechanical properties of GeTe in the temperature range 25–450 °C. To gain a better understanding of the mechanical behavior, elastic moduli were ultrasonically determined, and fracture surface analysis and diffraction methods were utilized, including electron backscatter diffraction (EBSD) and X-ray diffraction (XRD).

## 2. Experimental Procedure

### 2.1. Samples Fabrication

The GeTe samples were prepared by mixing high-purity (5N) Ge and Te at equal atomic weight. The mix was melted in an electric arc furnace. Later, the melt was sealed in an evacuated quartz ampoule under a 10^−6^ Torr vacuum and placed in a rocking furnace for 15 min at 1000 °C. After complete re-melting and homogenization, the quartz ampoule was water quenched, and the ingots were retrieved. The ingots were pulverized using a mortar and pestle and sieved using a 60-mesh sieve to achieve a particle size of 250 microns. The particles were hot-pressed at a pressure of 21 MPa and temperature of 550 °C. A total of three cylinders were fabricated (designated R1–R3). The resulting GeTe cylinders were polished up to 0.004 mm parallel surfaces with a height of ~13 mm. Finally, 5.8 mm-diameter samples were cut using an electric discharge machine (EDM). X-ray examination of the fabricated alloy reported in previous work [13] confirmed a single α-GeTe phase with *R3m* symmetry.

### 2.2. Ultrasonic Time-of-Flight

Elastic constants of the manufactured materials were evaluated via measuring the time of flight (TOF) of an acoustic wave to travel within the material—also known as the pulse-echo method. The results were obtained from the hot-pressed cylinders before EDM cutting for compression specimens. In this technique, a piezo-electric transducer employs a mechanical pulse on the sample surface, thus generating either longitude or a shear ultra-sonic wave in the sample. The return of these sound waves is detected by the same transducer and its amplitude was amplified. The frequency of both transducers was 5 MHz. With knowledge of the specimen geometry and using a digital oscilloscope, one can measure the time of flight of the sound waves between two successive echoes. All elastic constants can then be calculated using the equations given in ASTM D 2845 [15], with emphasis on Young’s modulus (E), Shear modulus (G), Poisson’s ratio (ν), and the Bulk modulus (B).

### 2.3. Compression Testing

**Figure 1:**
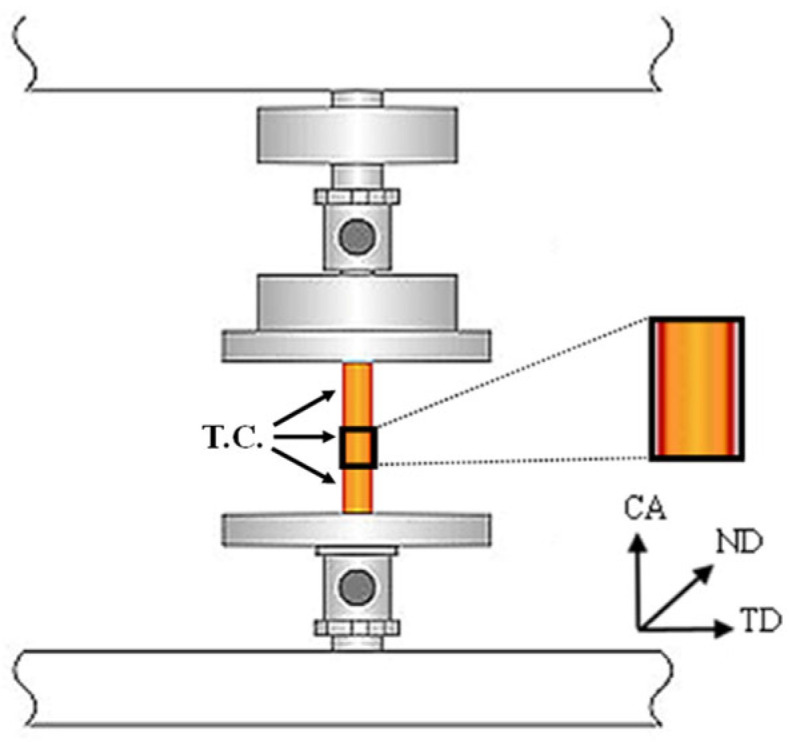
Schematic representation of the compression testing system. The notations CA, ND, and TD represent the compression, normal, and transverse directions, respectively. TC = thermocouple. The orange rectangle describes the GeTe rod.

The length and diameter of the samples were measured using a micrometer. A small amount of MoS_2_ lubricant was applied on the bases of the samples, and the samples were placed between the compression plates. A schematic representation of the compression testing system is presented in Figure 1. The notations CA, ND, TD, and TC depict the compression, normal, transverse directions, and thermocouple, respectively. An initial 20 MPa stress was applied and maintained while the sample was heated (at ~10 °C/min) and held at the testing temperature until stabilization of all apparatuses. The testing temperatures were room temperature (RT), 150 °C, 250 °C, 300 °C, 350 °C, 400 °C, and 450 °C. The sample was then loaded at a rate of 0.1 mm/min up 70 MPa and then released to 40 MPa for several cycles to estimate the compression modulus (all cycles were conducted in the elastic response region, so the material’s further mechanical response was not compromised). Finally, the sample was loaded at the same load rate up to fracture. The fracture surfaces following compression tests were studied using a standard stereoscope and a scanning electron microscope with a field-emission gun (FEG-SEM).

## 3. Results

### 3.1. Elastic Constants Determination Using TOF

The cylinder-shaped GeTe samples were tested for elastic constants, employing the ultrasound–time-of-flight (US-TOF) method.

Results are summarized in Table 1. The value of Young’s modulus obtained in the current research (59.1 ± 3.6 GPa) was similar to that reported in a recent study (56.1 ± 0.5 GPa, [16]). A higher Young’s modulus was obtained through ab-initio first principles calculations [17]. This difference between the experimental and the theoretical values of Young’s modulus might be reasoned by the lower relative density (ρ_rel_) of the samples tested in current research, which might be the result of insufficient degassing during vacuum stage of the sample preparation—while the theoretical density of GeTe value of 6.14 gr/cm^3^ was taken from the literature [18].

### 3.2. Mechanical Testing

**Figure 2:**
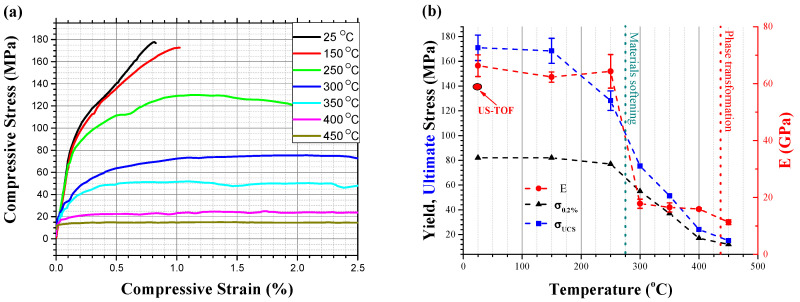
(**a**) Compression stress–strain curves and (**b**) mechanical and physical properties of GeTe at different compression temperatures.

Representative engineering stress–strain curves at different compression temperatures are given in Figure 2a.

Samples compressed at temperatures below 300 °C displayed a clear elastic region at low strains. At low compression temperatures (RT to 150 °C), plastic deformation was accompanied by deformation hardening before the final fracture, characteristics of a brittle fracture. At higher compression temperatures, starting at 250 °C, ductile characteristics were present, with a significant plastic flow that increased with increasing testing temperature. At temperatures above 400 °C, a perfectly plastic behavior was observed without any deformation hardening.

Figure 2b summarizes the compression characteristics as a function of temperature. With the raising of the testing temperature, a negative gradient change in the mechanical properties was noted. A rapid drop in mechanical properties could be seen at ~300 °C, well below the melting (625 °C) and phase transformation (430 °C) temperatures according to the Ge-Te equilibrium phase diagram [20].

All compression results of the characteristic specimens for each temperature are summarized in Table 2, where E is Young’s modulus, σ_0.2%_ is yield strength by 0.2% plastic strain offset, and σ_UCS_ is the ultimate compression strength.

### 3.3. Fracture Surface Characterization

**Figure 3:**
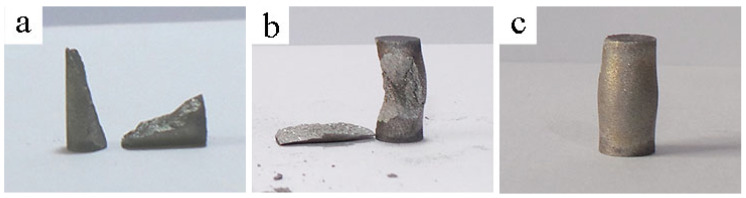
Low-magnification images of the specimens after compression at (**a**) up to 150 °C, (**b**) 250 °C, and (**c**) 350 °C.

Low-magnification images of the specimens after compression at RT, 250 °C, and 350 °C are presented in in Figure 3a, Figure 3b and Figure 3c, respectively. Based on the deformed structure of the samples post-compression, the samples could be separated into two testing groups. The 1st group includes severely fractured samples following hot-compression from RT up to 250 °C, whereas specimens in the 2nd group are those which experienced extensive plastic deformation without failure following hot-compression at temperatures 300 °C and above, see in Figure 3c. In the 1st group, samples compressed at RT and 150 °C fractured and split into two equal pieces under mode I loading (pure compression) as indicated by the cleavage plane aligned 45° to the compression axis. At 250 °C, the samples experienced slightly higher plastic deformation than those compressed at lower temperatures. Small and uneven parts fragmentation following compression for the 250 °C samples is attributed to macro-sized cracks that also propagated along the circumference, as shown in Figure 3b. The gradual shift from a catastrophic rapture to quasi-fracture and finely to a deformed state is consistent with the obtained compressive stress–strain curves, showing higher plasticity with increased temperature.

**Figure 4:**
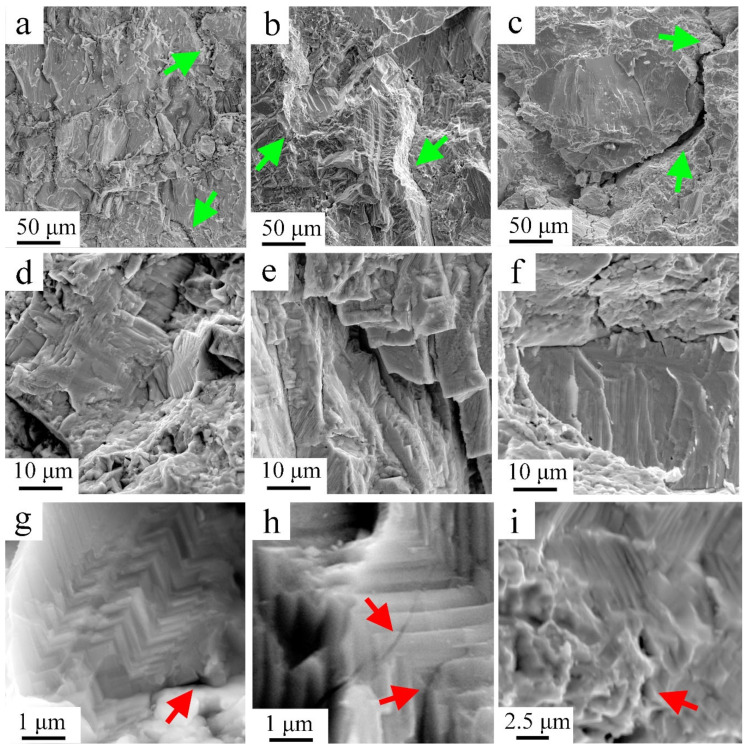
Fracture surfaces at different magnifications after compression at (**a**,**d**,**g**) RT, (**b**,**e**,**h**) 150 °C, and (**c**,**f**,**i**) 250 °C. Green and red arrows pointing to intergranular micro-cracking at different magnifications.

The fracture surfaces of the compression specimens are shown in Figure 4. The jagged-looking micro-cracks with sharp edges at the fracture surfaces point to intergranular cracking. For clarity, these features are presented in all the pictures but are pointed out using green arrows only in Figure 4a–c. Moreover, most of the fracture surface includes pure cleavage facets, i.e., without dimples (as can be seen with slightly higher magnifications in Figure 4d–f), thus pointing to the brittle nature of these samples under compression. These observations are in-line with the mechanical behavior of the 1st group, suggesting that fracture occurred before plastic yielding or after small plastic deformation. Higher magnifications of the cleavage facets revealed densely packed twins sorted in a zig-zag configuration with ~90°, see Figure 4g–i. That unique twinning configuration was reported in ref. [21]. There, it was reasoned by polymorphic phase transformation from the *Fm*-3*m* (cubic) phase to the *R*3*m* (rhombohedral) phase during cooling [22]. The twins’ interface gradually defused with increased compression temperature, as noticed in Figure 4g–i. Additionally, the micro-cracks propagated differently with testing temperatures (red arrows in Figure 4g–i). Following compression at RT, micro-cracks propagated strictly along the twins’ interfaces, as seen in Figure 4g, whereas at 150 °C, some of the micro-cracks also started crossing twins (Figure 4h). Supposedly, the subtle changes in the way micro-cracks propagate are related to the state of the twins. Further discussion on this is conducted later in this paper. It should be mentioned that at the higher compression temperature (250 °C), the fracture surface was much more chaotic due to the higher magnitude of deformation, and as such, it was impossible to differentiate cracks and learn about their nucleation or their prorogation path (Figure 4i).

As can be summarized for this sub-section, at low testing temperatures (under 150 °C)—where coherent high angle twin boundaries are presented—fracture occurs before plastic deformation (or with a little portion of it). The twin boundaries might act as barriers for dislocation movement, the plastic deformation is limited, and fracture advances along the twins and grains boundaries and on cleavage planes inside the grains. As the testing temperature rose, the interface between adjacent twins dissolved. Therefore, their efficiency as dislocation barrier decreased, which led to higher plastic deformation and fracture could advance between twins as well. The defused twin boundaries resulted in the overall increase of dislocation movement and the mode of fracture transformed from brittle to ductile.

To better understand the mechanical behavior of the GeTe alloy under hot compression, a series of compression tests were conducted to a stress value just above the yield stress (YS), at testing temperatures of 250 °C and 350 °C. Testing at these specific temperatures was based on the drastic changes in the mechanical properties at these compression temperatures. The tests were stopped just above the YS to enable microstructure analysis using scanning electron microscopy (SEM) to better characterize the deformation mechanism and the source of the initial plastic strain.

**Figure 5:**
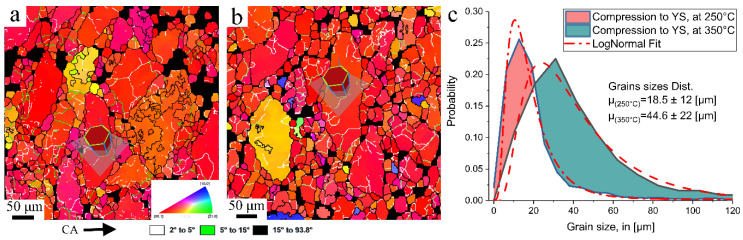
EBSD orientations maps (ND||z) of the hot-compressed GeTe samples to YS at (**a**) 250 °C and (**b**) 350 °C. A color scheme key in the form of the stereographic triangle is shown in (**a**). White, green, and black lines emphasize low, medium, and high grain-boundary misorientations. (**c**) Distribution of grain sizes. Samples compressed at 250 °C (red) and 350 °C (green).

Post-hot-compression testing, the samples were sectioned along the compression loading axis (CA), followed by traditional mechanical and chemical polishing procedures. The electron backscattered diffraction (EBSD) technique was utilized for micro-texture analysis and microstructure studies, including statistical characteristics of dislocations density (ρ). The ATEX package was employed for post-processing and visualizing the EBSD data [23]. Figure 5a,b present color-coded orientation maps along the CA-TD plane respectively. Imposed on these maps is the grain boundaries (GBs) information, which was derived based on the misorientation angle criteria, with low (white lines), medium (green lines), and high (black lines)—designated by 2 to 5°, 5° to 15°, and 15° to 93.8°, respectively.

The microstructure of the hot-compressed specimens comprises coarse and fine-sized grains. The coarse grains are mostly described by their asymmetric shape, with a short axis parallel to the CA. The fine grains are packed into colonies or laced peripherally along the coarse-sized grains. Twins were not seen in the EBSD maps, probably due to their nano-size structure (Figure 4). According to the inverse pole figure (IPF) maps, both samples presented strong preferential micro-texture where most grains had their basal planes parallel to the CA, or *c*-axes perpendicular to the CA. Under close examination, a color gradient was not observed in the core of the grains for both testing conditions. Noting that a grain’s color represents an orientation, the lack of color gradient plausibly implies low disorientation (i.e., low plastic strains) throughout the inner structure of each grain. That is reasoned with the testing conditions which, as mentioned, included a compression test just above the YS.

Figure 5c summarizes grain-size measurements made based on the grain-reconstruction method [24]. Comparing both distributions, there seems to be a notable shift to higher values of grain sizes with testing temperature. These changes are attributed to structural modifications which involve a gradual increase in the nominal grains size (both the fine and the coarse-sized grains) with compression temperature. It is likely that a coarsening of the grains was a direct outcome of temperature and probably an indirect outcome of plastic stress. Both concepts are now thoroughly investigated using quantitative analysis focused on the dislocations activity as inflected by the testing conditions.

**Figure 6:**
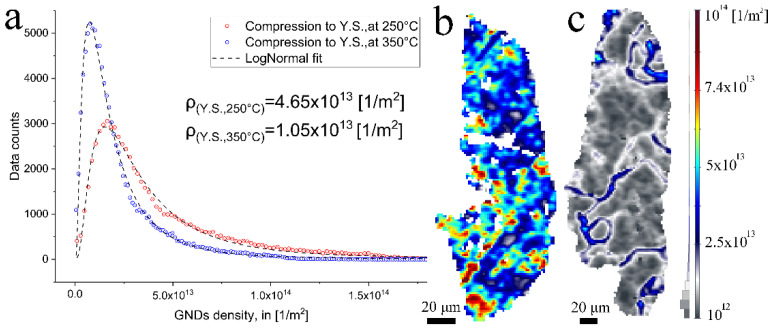
(**a**) Distribution of discrete GNDs and (**b**,**c**) GNDs maps for segmented grains in the hot-compressed specimens to YP at 250 and 350 °C. A color scheme key for GNDs density maps is shown in (**c**).

The plasticity of a material can be addressed through quantitative analysis of dislocation density. Following works by Nye [25] and Pantleon [26], the total dislocation density is composed of two types of dislocations: Geometrically Necessary Dislocations (GNDs) and Statistically Stored Dislocations (SSDs). Unlike the SSDs, the GNDs’ movement results in localized misorientations in a form of lattice curvatures between spatially separated neighboring measurements. As the EBSD technique is sensitive to localized misorientations, the GNDs can be extracted by calculating the curvature components [27]. Figure 6a presents the distribution of the discrete GNDs, as calculated from the EBSD data (selected grains marked with hexagons in Figure 5a,b). The discrete GNDs in both cases follow a lognormal distribution; hence, each grain-averaged GNDs density ($m\left(\rho \right)$) was calculated based on a lognormal fitting, using the following equations.

(1) $$f\left(\rho |\mu ,\sigma \right)=\frac{1}{x\sigma \sqrt{2\pi }}exp\left(\frac{-{\left(\ln\left(\rho \right)-\mu \right)}^{2}}{2\sigma^{2}}\right)$$

(2) $$m\left(\rho \right)=exp\left(\mu +\frac{\sigma^{2}}{2}\right)$$

where *µ* and *σ* are the location and scale parameter of the lognormal distribution, and *m*(*ρ*) is the mean value of *ρ*. It turns out that the GNDs density significantly decreased with temperature testing, from 4.65 × 10^13^ to 1.05 × 10^13^ (1/m^2^). The change in GNDs density was evaluated using GNDs density maps, providing spatial information on the distortions of the crystal lattice as introduced by hot-compression up to the YP at 250 °C and 350 °C, respectively. The maps were plotted specifically for two representative grains, chosen based on their similar orientation and size, and they were marked using lattice cells as shown in Figure 5. The GNDs density maps for these grains are presented in Figure 6b,c. At the lower compression temperature, the GNDs are spread heterogeneously with multiple peaks near the grain boundary edges and at the grain core. At the higher compression temperatures, non-uniform distribution of the GNDs was noticed. The GNDs are arranged in dense configurations paved from the grain boundary to its core. Plausibly, these energetically favorable configurations are the starting point in the fragmentation of grains into sub-grains.

The decrease in GNDs density and their unique arrangement might be attributed to the dislocation–twin interactions. Recalling that the fracture surface at the lower testing temperatures included densely packed twins, in Figure 4a,b,d,e, and that the twin’s interfaces defused to a higher extent with temperature (in Figure 4c,f). Plausibly, the twins’ interface, when it preserves coherency with the matrix, acts as an effective barrier to dislocation glide, thereby resulting in a significant strain hardening, as interpreted by the high ratio of UCS to YS at the lower testing temperatures, see Figure 2b. In turn, the higher GNDs density at lower testing temperatures could be reasoned by entangled and piled-up dislocations at the twins’ interfaces. Under the combined effects of higher temperature (350 °C) and compression, the coherency of the twins gradually deteriorated, resulting in their partial consumption, as observed by their diffused interface, see Figure 4i. With a “twin-free” state, dislocation mobility substantially increased. Thereby, further plastic deformation was assisted by higher dislocation activity, resulting in annihilation and their arrangement to densely packed dislocation walls. The above description aligns with the lower YS and increased plasticity found at the higher compression temperature, as seen in Figure 2, and GNDs analysis.

## 4. Discussion

The Current research set out to investigate temperature-dependent mechanical properties of the thermoelectric GeTe compound. The GeTe compound is characterized by a melting temperature of 625 °C. Phase transformation from the rhombohedral structure (α-phase) to the cubic (β-phase) occurs at ~427 °C (0.78T_H_, where T_H_ represents the homologous temperature). In the current study, it was shown that the overall mechanical behavior of the GeTe shifted from brittle to ductile at ~300 °C (which makes around 0.64 T_H_). Such a rapid change in the mechanical behavior of materials might be the result of local structural changes, but is usually attributed to changes in deformation mechanisms. Such mechanical degradation under uniaxial hot compression was also observed at ~0.45 T_H_ for the nanostructured (Bi_0.2_Sb_0.8_)_2_Te_3_ [28]. In the same research, Lavrentev et al. proposed grain boundary slippage as the dominant mechanism in the nanostructured samples for the sudden drop in strength with temperature. In the current study, samples had grain sizes on the microscale, and in turn, deformation was assisted by dislocation slip and pile-up within the grains. Generally, dislocations move more “easily” on preferred slip systems with low critical resolved shear stress (CRSS). Their movement might be hindered by interfaces such as high angle boundaries and twins, and in turn results in complex dislocation configurations of localized pile-ups with high strain fields. Applying further external energy such as heat, during mechanical loading, the driving force for microstructural changes such as boundaries dissolving and/or dynamic recrystallization is increased. The GNDs map shown in in Figure 6b,c supports the theory of dislocation slippage and pile-ups. It shows that with increasing testing temperature the GNDs density, and therefore the stored energy within the grains, is decreased.

Reviewing the fracture surfaces as seen in Figure 4, it is shown that with increasing compression temperature the fracture transforms from brittle intergranular with a significant presence of twinning at room temperature up to 150 °C, to a more ductile intra-granular fracture with much less evidence of twinning at temperatures higher than 250 °C. Furthermore, with increasing temperature, the twins’ boundaries seem to dissolve, which means higher dislocation mobility across adjacent twins and, therefore, higher plastic deformation.

**Figure 7:**
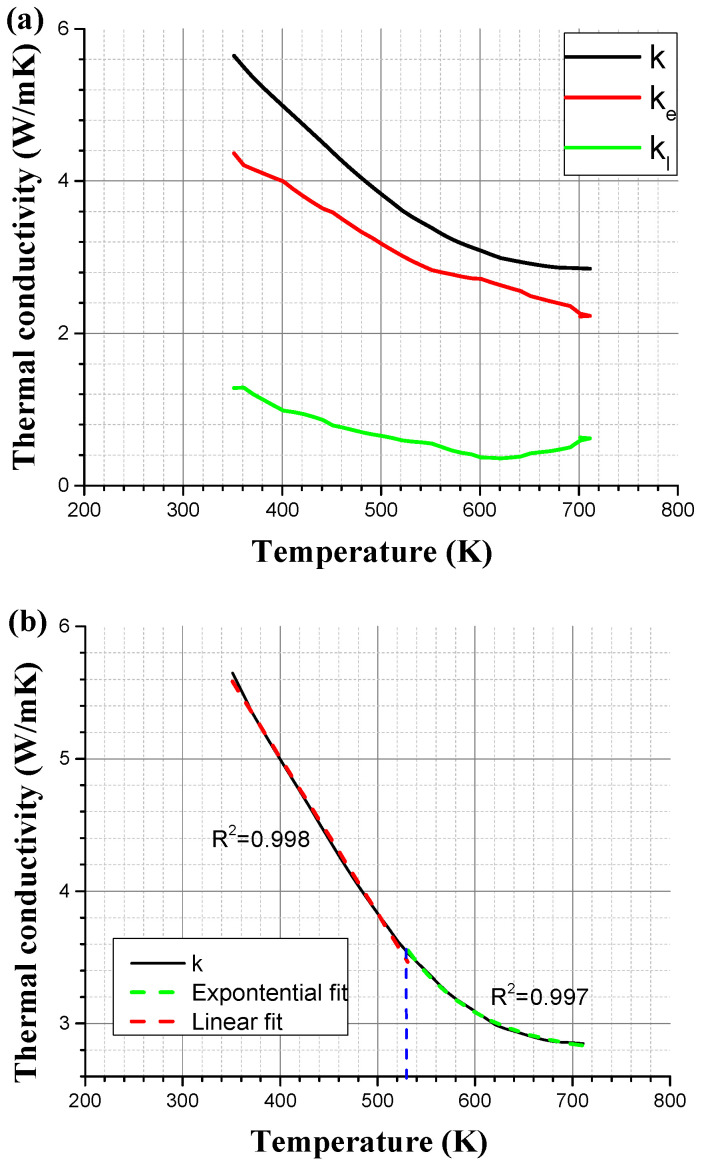
Thermal conductivity of GeTe. (**a**) Divided to electrons and phonons conductions, (**b**) linear and exponential trends intersection [13].

The cubic-to-rhombohedral structure phase transition during cooling results in the formation of domains constructed of multiple thermal twins. These domains contribute to an increase of inherent defects density in the microstructure [29] and thus higher phonon scattering. Therefore, a lower thermal conductivity is presented, which sums up an overall higher figure of merit [30]. A re-examination of the transport properties of the manufactured GeTe [13] was conducted to evaluate the electronic (κ_e_—red line) and phonon (κ_l_—green line) contributions to the total thermal conductivity (κ—black line), which has was explained in previous work [31] and can be seen in Figure 7a. It seems that for the electronic contribution, there is a constant linear relation with raising the temperature, while the phonon contribution is quite constant, with a slight increase at around 300 °C. The overall thermal conductivity seems to have a transition from linear relation into a more subtle exponential relation at roughly 260 °C (~530 K), which is a direct result of the change in the phonon contribution, as can be seen in see Figure 7b. This change is in-line with the diffused twins’ boundaries as described above. Twin boundaries act as phonon scatters and reduce their contribution to thermal conductivity. A decrease in the twin boundaries density will lead to a decrease in phonon scattering, which will result in an increase or preservation of thermal conductivity. Such change in thermal conductivity will eventually result in a lower figure of merit (*ZT*) at raising temperatures.

## 5. Conclusions

The main goal of this study was to study the thermo-mechanical response of the thermoelectric GeTe compound under compression and its deformation mechanisms. Additionally, a detailed investigation of the crystal structure, using a combination of experimental and computational methods, was carried out. The compression tests demonstrated a shift from brittle to ductile behavior at a temperature of ~250 °C. Furthermore, the fracture surface at low and high compression temperatures demonstrated the transition from trans-granular and along the twins’ interfaces at low temperatures to intergranular fracture at high temperatures due to extensive plastic deformation change. At lower testing temperatures, a high density of dislocations piled up at twins’ interfaces exists. With increasing testing temperatures, the coherency of the twins’ interface degraded, thus enabling extensive plasticity by higher dislocation mobility. This hypothesis of the twins’ boundaries dissolvent was also motioned by an increase in phonon contribution to the overall thermal conductivity, which was also shifted at ~250 °C. This research shows that although many thermoelectric alloys and compounds, such as GeTe, exhibit brittle mechanical nature at room temperature, they might exhibit a much more preferable ductile nature at higher operating temperatures.

## Figures and Tables

**Table 1:** Elastic constants values of GeTe that were obtained in this work and refs. [13,19].

References	Young’s Modulus (E) (GPa)	Shear Modulus (G) (GPa)	Bulk Modulus (B) (GPa)	Poisson’s Ratio (ν)	Relative Density (ρ_rel_) (%)
This work	59.1 ± 3.6	23.4 ± 1.6	42.4 ± 0.9	0.27 ± 0.01	98.5
J. Davidow et al. [16]—TOF	56.1 ± 0.5	---	---	---	98.78
H. L. Kagdada et al. [17] based on first principles calculation	67.14	28.16	36.31	0.19	100

**Table 2:** Mechanical properties under compression at temperature-dependent results.

Temp. (°C)	E (GPa)	σ_0.2%_ (MPa)	σ_uCS_ (MPa)
25	66 ± 4	82 ± 5	171 ± 10
150	62 ± 2	82 ± 7	169 ± 10
250	64 ± 6	77 ± 6	128 ± 8
300	18 ± 2	55 ± 4	75 ± 6
350	17 ± 2	37 ± 4	51 ± 2
400	16 ± 1	17 ± 3	24 ± 1
450	11 ± 1	12 ± 3	15 ± 1
